# Vascular Smooth Muscle Cell Metabolic Reprogramming in Arteriovenous Fistula Failure

**DOI:** 10.3390/biomedicines13102340

**Published:** 2025-09-25

**Authors:** Jingpeng Bao, Guiqing Tian, Yuchi Tu, Qianqian Liao, Lijun Yao

**Affiliations:** 1 Department of Nephrology, Union Hospital, Tongji Medical College, Huazhong University of Science and Technology, Wuhan 430022, China; 2 Wuhan Clinical Research Center for Metabolic Chronic Kidney Disease, Department of Nephrology, Wuhan Fourth Hospital, Wuhan 430030, China

**Keywords:** vascular smooth muscle cell, metabolic reprogramming, arteriovenous fistula dysfunction, therapy

## Abstract

Hemodialysis is the most commonly used renal replacement therapy worldwide, and arteriovenous fistulas (AVFs) are the preferred vascular access. The functional patency of AVFs directly determines the dialysis efficiency and quality of life of patients with end-stage renal disease (ESRD). However, in clinical practice, the failure of AVFs seriously affects the treatment prognosis of these patients. Vascular smooth muscle cells (VSMCs), as the main component of the vascular media, not only maintain the integrity and tension of the vascular wall under physiological conditions but also play a crucial role in the failure of AVF maturation and post-maturation dysfunction. VSMCs undergo metabolic reprogramming along with adaptive structural and functional alterations, driven by persistent stimulation from hyperphosphatemia, uremic toxins, oxidative stress, and inflammatory factors, as well as hemodynamic disturbances induced by AVF creation and surgical trauma. This review summarizes the roles of VSMC metabolic and phenotypic shifts in outward remodeling, inward remodeling, and medial calcification during AVF failure, elaborates on the metabolic crosstalk between VSMCs and endothelial cells, and discusses potential therapeutic targets targeting VSMC metabolism.

## 1. Introduction

Chronic kidney disease (CKD) is a serious public health issue. Currently, there are 697.5 million CKD cases worldwide, with a global prevalence rate of 9.1% [1], and over 2.6 million people globally receiving renal replacement therapy [2]. Due to the problems of obtaining and using kidney sources in kidney transplantation, dialysis is still the main treatment method in most countries, and hemodialysis is the most commonly used dialysis modality [3]. Compared with arteriovenous grafts (AVGs) and central venous catheters (CVCs), arteriovenous fistulas (AVFs) are recognized as the preferred vascular access for most hemodialysis patients due to their durability, lower infection risk, and reduced need for interventions [4]. A recent multicenter prospective cohort study (*n* = 7) showed that the 1-year functional patency rate of mature AVFs was 87% and the 2-year rate was 75%. Among mature AVFs, nearly half (188 cases, 47.5%) required further intervention to maintain patency or treat complications [5]. Although AVFs are regarded as the optimal vascular access for hemodialysis, they still fail to meet the clinical needs of permanent treatment. Therefore, the mechanisms of AVF dysfunction are worthy of further study.

As the main component of the vascular media, vascular smooth muscle cells (VSMCs) are involved in maintaining vascular wall integrity and tension. Interestingly, even in adult organisms, VSMCs still possess plasticity and can undergo significant phenotypic changes in response to changes in local environmental signals [6]. This property plays an important role in many vascular-related diseases such as atherosclerosis [7], pulmonary hypertension [8,9], and hypertension [10]. This pathophysiological mechanism also contributes to outward remodeling, inward remodeling, and vascular calcification in AVF dysfunction.

Metabolism is a fundamental biological process in living cells. Numerous experimental findings have demonstrated that the metabolism of VSMCs plays a crucial role in vascular-related diseases [11]. More importantly, studies have revealed that following AVF creation, differentially expressed mRNAs in the vascular tissue at the anastomotic site are involved in multiple metabolic processes and pathways [12]. This indicates that the metabolism of VSMCs also influences AVF function. Metabolic reprogramming refers to the dynamic alteration of cellular metabolic pathways to meet bioenergetic and biosynthetic demands under physiological or pathological stress, enabling functional adaptation [13]. VSMCs are not only continuously stimulated by hyperphosphatemia [14], uremic toxins [15], and inflammatory factors [16] in the CKD state, but also need to cope with hemodynamic abnormalities [14] caused by AVF creation. The metabolic and phenotypic remodeling they undergo to adapt to these pathophysiological factors ultimately accelerates the progression of AVF dysfunction. This review aims to clarify the specific regulatory mechanisms of metabolic reprogramming and phenotypic switching of VSMCs in the process of AVF dysfunction and systematically summarize the research progress of potential therapeutic drugs targeting the abovementioned metabolic abnormalities.

## 2. Pathophysiological Features of Arteriovenous Fistula Dysfunction

AVF creation entails the direct anastomosis of an artery to a vein [17]. Following AVF creation, the fistula vessel undergoes inward and outward remodeling [18], and imbalance in vascular remodeling leads to AVF dysfunction. Furthermore, medial vascular calcification is one of the key factors contributing to AVF dysfunction. Therefore, this part will briefly describe the pathophysiological characteristics of AVF dysfunction from three aspects mentioned above.

After the formation of AVF, the contractile VSMCs change to a synthetic phenotype. Contractile VSMCs express α-smooth muscle actin (SMA-α), myosin heavy chain 11 (MYH11), and SM22α. This switch is characterized by decreased expression of contractile markers and increased secretion of extracellular matrix (ECM), matrix metalloproteinases (MMPs), and proinflammatory cytokines. In addition, VSMCs can exhibit phenotypes similar to other cell types, including osteogenic/chondroid VSMCs (expressing OPN and RUNX2) [19,20,21].

### 2.1. Outward Remodeling

In Kidney Disease Outcomes Quality Initiative (KDOQI) 2019, AVF functional maturity is defined as the ability to provide a prescribed dialysis consistently with two needles for more than two-thirds of the dialysis sessions within 4 consecutive weeks, as well as ensuring that vessel diameter > 5 mm and blood flow > 500 mL/min are satisfied [22]. The creation of an AVF results in increased blood flow, blood volume, and shear stress in the outflow vein. The vessel must undergo outward remodeling to enable the outflow vein to withstand the elevated pressure [23]. Outward remodeling relies on a delicate balance of extracellular matrix (ECM) remodeling, VSMC proliferation, inflammation, growth factor secretion, and upregulation of cell adhesion molecules in the venous wall to promote “venous arterialization,” which is characterized by luminal dilation and wall thickening, and accommodate the increased blood flow induced by dialysis [24,25,26]. In a previous study, researchers used a novel VSMC lineage-tracing reporter mouse to establish an AVF model. The researchers observed medial VSMC layer thickening in the mid-segment of the arterialized venous branch at 4 weeks post-surgery, with this layer composed entirely of differentiated MYH11+/Ki67- cells. Notably, identical processes were recapitulated in human samples [27]. In RP105 -/- mice with AVF, VSMCs shift toward a regenerative phenotype with reduced proliferation, resulting in impaired venous outward remodeling [28]. CKD-induced oxidative stress results in NO resistance of the NO receptor sGC in VSMCs, leading to impaired vascular relaxation and thus hindering AVF maturation [29]. In summary, the proliferation of differentiated contractile VSMCs in the vascular media is indispensable for outward remodeling.

### 2.2. Inward Remodeling

Excessive inward remodeling, also known as neointimal hyperplasia, not only leads to AVF maturation failure (accounting for approximately 60% of cases) but also induces dysfunction in mature AVFs [18,30]. These processes can ultimately promote excessive inward remodeling. It typically occurs in the venous segment near the anastomosis [31] and juxta-anastomotic area (JAA) [32]. Compared with the outflow vein, the JAA has a greater volume of disordered blood flow. The endothelial cells on the inner wall of the blood vessel on the opposite side of the fistula outlet will be rapidly lost, which will subsequently form a thrombus and eventually lead to neointimal hyperplasia [32,33]. Excessive intimal hyperplasia leads to venous outflow tract stenosis, reduced fistula blood flow, and significantly decreased suitability of the fistula for dialysis [34]. To make matters worse, extensive wall shear stress fluctuations develop distal to the stenotic segment, which damages endothelial cells and exacerbates intimal hyperplasia, thereby establishing a vicious cycle [32,35,36]. For AVF stenosis with obvious clinical and angiographic manifestations, guidelines recommend high-pressure balloon angioplasty as the first-line treatment [22]. However, AVFs are highly prone to restenosis, and the durability of percutaneous transluminal angioplasty is limited, with patency rates ranging from 26% to 64% at 12 months after surgery [37]. Thereby fistula resources in MHD patients are gradually depleted.

The artery of an AVF is the main source of smooth muscle cells in the process of neointimal formation [38]. Hemodynamic stress and mechanical injury can cause damage to endothelial cells and smooth muscle cells, thereby promoting the migration of smooth muscle cells from the vascular media to the intima, where they proliferate and differentiate into a secretory phenotype [39]. Patients with CKD exhibit systemic inflammation [40], which persists throughout the entire lifespan of the AVF. This inflammatory state largely shapes the AVF microenvironment [18,40], which can influence the phenotypic switch of VSMC. In vitro experiments by Rai et al. demonstrated that macrophages and pro-inflammatory cytokines (IL-6, IL-1β, and TNF-α) can promote the transition of VSMCs to a dedifferentiated phenotype, impeding AVF maturation [16]. Elevated PTH upregulates the myofibroblast marker integrin β6 (ITGB6) via the phosphorylated Akt pathway, promoting VSMC transdifferentiation into myofibroblasts and augmenting the risk of AVF maturation failure [41].

A recent study revealed that the dynamic changes in VSMC phenotype are central to AVF maturation. Following AVF creation, VSMCs first dedifferentiate from the contractile to the synthetic phenotype, and then redifferentiate into the contractile phenotype at a later stage. Sustained activation of the Notch signaling pathway in VSMCs promotes neointimal formation, whereas the absence of Notch signaling during the early stage of AVF remodeling (before AVF surgery or within 1 week postoperatively) prevents the accumulation and differentiation of VSMCs, thereby failing to form functional AVFs [42]. This indicates that the processes of outward remodeling and inward remodeling involving VSMCs are not completely opposing aspects; instead, they can be linked through certain molecular pathways.

### 2.3. Medial Calcification

Numerous studies have demonstrated an association between vascular calcification and arteriovenous fistula dysfunction [43,44,45]. Vascular calcification can occur in the intimal layer or medial layer. Intimal calcification mainly occurs in atherosclerotic plaques, is associated with VSMCs and macrophages, and easily causes plaque rupture [46,47]. Medial calcification, also known as Monckeberg sclerosis, is common in patients with diabetes and CKD. It is characterized by the formation of amorphous minerals along or in the circumference of one or more elastic lamellae in the medial layer [48]. The radial artery is the preferred artery for AVF creation, and radial artery calcification in patients with end-stage renal disease (ESRD) mainly occurs in the medial layer [49]. After the establishment of an arteriovenous fistula, the increased blood flow induces elevated wall shear stress, which suppresses intimal hyperplasia and drives outward vascular remodeling. Medial calcification prior to AVF creation increases vascular stiffness, prevents the aforementioned vascular remodeling, and ultimately leads to AVF dysfunction [50,51].

Uremic toxins, hyperphosphatemia, calcium overload, and fluid shear stress in maintenance hemodialysis (MHD) patients have all been confirmed as stimulatory factors for medial vascular calcification [14,52,53,54]. In addition, MHD patients commonly suffer from Malnutrition–Inflammation Complex Syndrome [55]. Clinical studies have confirmed that malnutrition is a risk factor for vascular calcification in MHD patients [56,57]. After adjusting the dietary protein composition ratio from 25% to 2.5% in uremic rats with medial calcification, the incidence and severity of medial calcification increased significantly, indicating that a very-low-protein diet induces medial calcification [58].

For many years, vascular calcification was regarded as a passive process caused by elevated serum phosphate (P^2−^) levels and an increased calcium–phosphate product (Ca^2+^ × P^2−^), which leads to plasma supersaturation. Recent studies have demonstrated that vascular calcification is an active, tightly regulated osteogenic process primarily driven by VSMCs [48,59,60]. In the setting of inadequate responsiveness to pro-calcific stimuli, osteogenic/chondrogenic transdifferentiation of VSMCs is triggered. This cascade subsequently elicits the expression of downstream osteogenic mediators, such as type I collagen, alkaline phosphatase, osteopontin, and osteocalcin [61]. Our team has previously confirmed that SGK3 can enhance the expression and activity of Pit-1, ultimately promoting hyperphosphatemia-induced phenotypic switching of VSMC and vascular calcification in CKD [62].

Transdifferentiated VSMCs secrete matrix vesicles containing apoptotic bodies, necrotic fragments, endosomes, autophagosomes, and enriched alkaline phosphatase (ALP), which serve as sites for hydroxyapatite crystal precipitation [61,63,64]. These matrix vesicles can be endocytosed by adjacent normal VSMCs and accelerate the calcification process [65]. In addition, VSMCs express MMP-3, which degrades elastin. Inducing calcium deposition on fragmented and degraded elastin serves as an important promoting factor for medial calcification [66,67]. The reduction of calcification inhibitors matrix GLA protein (MGP) and fetuin A can be observed in patients with CKD [60]. VSMCs can synthesize MGP, which partially inhibits the osteoinductive activity of BMP-2 [68]. Fetuin A, synthesized by the liver, can chelate calcium and phosphate. It is readily absorbed by VSMCs and concentrated in internal vesicles, where it inhibits nucleated calcium phosphate precipitation [69].

## 3. VSMC Metabolic Reprogramming and Outward Remodeling

The central role of VSMCs in outward remodeling is reflected in the proliferation of differentiated contractile VSMCs in the media. Studies have shown that VSMCs undergo dynamic changes in phenotype during AVF maturation: VSMCs first dedifferentiate from a contractile phenotype to a synthetic phenotype, and then redifferentiate to a contractile phenotype at a later stage [42]. This suggests that the restoration of the contractile phenotype in VSMCs may be a factor contributing to vascular outward remodeling. Prohibitin 2 directly interacts with hnRNPA1 via its C-terminus, antagonizing the hnRNPA1-mediated alternative splicing of PKM mRNA and the switch from PKM1 to PKM2, thereby inhibiting enhanced glycolysis. Lkb1, on the other hand, reduces the PKM2/PKM1 ratio by regulating Ptbp1-dependent alternative splicing of PKM and inhibits the transdifferentiation of VSMCs into fibroblast-like cells. Both maintain the contractile phenotype of VSMCs by regulating the alternative splicing of PKM isoenzymes [70,71]. In summary, current research on the association between VSMC metabolic reprogramming and vascular outward remodeling remains limited, and this field is expected to be a worthwhile direction for in-depth exploration in the future.

## 4. VSMC Metabolic Reprogramming and Inward Remodeling

### 4.1. Glucose Metabolism

Under resting conditions, up to 45.5% of ATP in VSMCs is derived from glycolysis [72]. This metabolic feature is similar to the Warburg effect observed in cancer cells [73]. Accumulating evidence indicates that glycolysis in VSMCs is further upregulated during neointimal hyperplasia.

**Figure 1:**
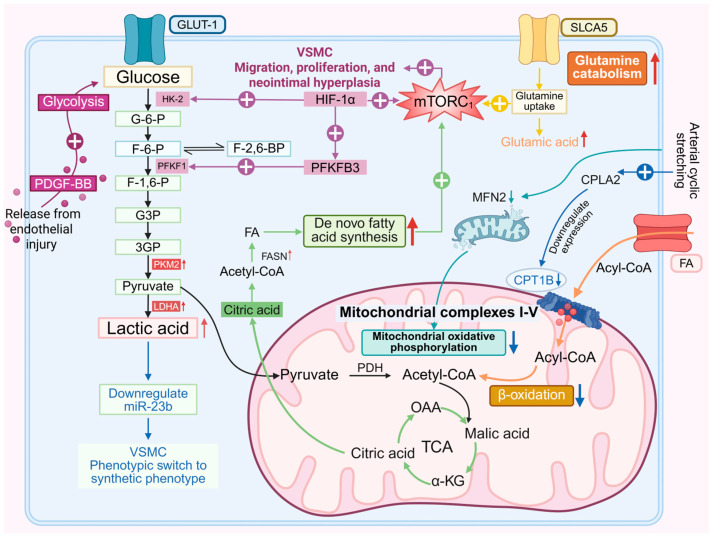
Vascular smooth muscle cell (VSMC) metabolic pathways, such as glycolysis, oxidative phosphorylation, fatty acid β-oxidation, de novo fatty acid synthesis, and glutaminolysis, are all implicated in arteriovenous fistula (AVF) neointimal hyperplasia. Glucose-6-phosphate (G-6-P); Fructose-6-phosphate (F-6-P); Fructose-1,6-bisphosphate (F-1,6-P); Fructose-2,6-bisphosphate (F-2,6-P); Glyceraldehyde-3-phosphate (G3P); 3-phosphoglycerate (3GP); Hexokinase 2 (HK-2); Phosphofructokinase-1 (PFKF1); 6-phosphofructo-2-kinase/fructose-2,6-bisphosphatase 3 (PFKFB3); Hypoxia-inducible factor-1α (HIF-1α); Pyruvate kinase M2 (PKM2); Lactate dehydrogenase A (LDHA); Glucose transporter 1 (GLUT-1); Tricarboxylic acid cycle (TCA); Oxaloacetic acid (OAA); α-ketoglutarate (α-KG); Fatty acid (FA); Carnitine palmitoyltransferase 1B (CPT1B); Cytosolic phospholipase A2 (CPLA2); Mitofusin 2 (MFN2); Solute carrier family A member 5 (SLCA5); Mammalian target of rapamycin complex 1 (mTORC1).

PDGF-BB released from the damaged intima induces phenotypic switching, proliferation, and migration of VSMCs (Figure 1), which is a key mechanism promoting intimal hyperplasia [74,75]. Glucose uptake, hexokinase 2 (HK2), liver-type phosphofructokinase 1 (PFKL), and lactate dehydrogenase A (LDHA) are all significantly upregulated in PDGF-BB-stimulated VSMCs [76,77]. After PDGF-BB treatment, crotonylation of PKM2 at K305 is upregulated in VSMCs, promoting PKM2 nuclear translocation. It interacts with STAT3 and β-catenin, regulating the transcription of MEK5, cyclin D1, glucose transporter 1 (GLUT1), and LDHA, thereby promoting aerobic glycolysis, SMC proliferation, migration, transition to a synthetic phenotype, and neointimal hyperplasia [78,79]. PDGF-BB can reduce the level of COX5A in mitochondria, alter its distribution, and decrease complex IV activity, ATP synthesis, and OCR, while increasing H_2_O_2_ synthesis, reactive oxygen species (ROS) production, and the ability of cell proliferation and migration [80].

Upregulation of the HIF-1α-PFKFB3 pathway in neointimal VSMCs leads to increased glycolysis and decreased contractile phenotype and promotes VSMC proliferation and activation of the mechanistic target of rapamycin complex 1 (mTORC1) [81]. Crotonylation of LDHA at lysine 5 activates LDHA by forming tetramers, and monoubiquitination at K76 induces LDHA translocation into mitochondria to promote mitochondrial fission; both enhance lactate production and VSMC proliferation and migration [82].

VSMCs are more prone to switch to the synthetic phenotype in a lactate-rich environment. The expression of bone morphogenetic protein-4 (BMP-4) is increased during intimal hyperplasia, which mediates lactate excretion through SMAD-4/MCT-4 signaling pathway and increases lactate in the microenvironment [83]. Lactate promotes the conversion of VSMCs to a synthetic phenotype by down-regulating miR-23b by targeting SMAD3 [84].

After the generation of an AVF, the outflow vein is suddenly exposed to arterial stress, which results in a decrease in mitochondrial fusion protein 2 (MFN2), mitochondrial fragmentation, and increased PFK1 stability, thereby shifting the metabolic pattern from mitochondrial oxidative phosphorylation to glycolysis [85]. Another study reached a similar conclusion, finding that there was abnormal proliferation and migration of VSMCs in patients with AVF stenosis, accompanied by increased mitochondrial fission [86].

### 4.2. Lipid Metabolism

Compared with venous stretch at 5%–1 Hz, arterial cyclic stretch at 15%–1 Hz increases long-chain fatty acid (LCFA) content in VSMCs. CPT1B, a key enzyme in fatty acid β-oxidation, is inhibited by high-intensity cyclic stretch via the cPLA2/YY1/CPT1B pathway under arterial pressure, and arterial pressure can promote VSMC proliferation, migration, and neointimal hyperplasia by inhibiting fatty acid β-oxidation [87]. FASN (fatty acid synthase) is upregulated in neointimal VSMCs of human carotid artery stenosis lesions. PFKFB3-mediated glycolytic reprogramming and FASN-mediated lipid metabolic reprogramming are characteristic events in VSMC phenotypic switching, and both drive neointimal formation via the acetyl-CoA/mTORC1 pathway [81]. Elovl6, a rate-limiting enzyme for LCFA elongation, drives VSMC phenotypic switching through ROS production and activation of the AMPK/KLF4 pathway [88].

### 4.3. Amino Acid Metabolism

Both TEAD (TEA domain transcription factor) 1 and 5-HT receptor 2B (5-HT2BR) are upregulated in injured arteries [89,90]. TEAD activates the mTORC1 signaling pathway by transcriptionally inducing the glutamine transporter SLC1A5 (solute carrier family 1 member 5), thereby promoting VSMC proliferation and intimal hyperplasia [89]. 5-HT2BR mediates the migration of rat aortic smooth muscle cells in a β-arrestin2-dependent manner via activation of the mTOR/p70S6K signaling cascade [90]. PDGF-stimulated VSMCs exhibit enhanced glutaminolysis to meet their increased energy demands and biomass synthesis [91].

## 5. VSMC Metabolic Reprogramming and Medial Calcification

### 5.1. Glucose Metabolism

#### 5.1.1. High-Phosphate Environments and VSMC Glucose Metabolism in Medial Calcification

Recent studies have demonstrated that in VSMCs with calcification induced by a high-phosphate environment, the expression levels of GLUT1, PKM2, PDK4, PFKFB3, LDHA, monocarboxylate transporter 1 (MCT1), and MCT4 are all upregulated, with a significant increase in lactate content [92,93,94] (Table 1).

In the CKD-related vascular calcification model, ovarian tumor ubiquitin-like protein 2 (OTUB2) promotes the binding of the YAP-TEAD1 complex to the PFKFB3 promoter, enhancing PFKFB3 transcriptional activity [99]. PFKFB3-mediated glycolysis drives osteogenic transdifferentiation of VSMCs by upregulating FoxO3 and promoting lactate production [95]. The orphan nuclear receptor NR4A3 (nuclear receptor subfamily 4 group A member 3) enhances glycolytic activity by directly binding to the ALDOA gene and PFKL promoter. NR4A3-mediated histone lactylation upregulates Phospho1 gene expression, promoting medial vascular calcification [100]. PDK4 can drive metabolic reprogramming of VSMCs, upregulating their shift to the Warburg effect; inhibiting PDK4 can block VSMC calcification [92,94]. PDK4 can enhance the BMP2 signaling pathway through SMAD1/5/8 phosphorylation, promoting osteogenic differentiation of VSMCs [92] PDK4 can inhibit the interaction between V-ATPase and lactate dehydrogenase B and nuclear translocation of transcription factor EB, reduce lysosomal degradation function, interrupt autophagic flow, and accelerate calcium deposition in VSMC. It was also found that 2-deoxy-D-glucose could induce VSMC apoptosis and increase calcium deposition despite its inhibition of glycolysis [94]. A review proposed the following hypothesis: this enhanced glycolysis may be an adaptive or protective mechanism of VSMCs in response to energy depletion or oxidative stress caused by pathogenic factors [73].

Some studies have shown divergent views on the impact of high-phosphate environments on VSMC glucose metabolism. Primary human aortic VSMCs treated with β-glycerophosphate (β-GP) exhibit a more oxidative and less glycolytic phenotype, with impaired ability to cope with stress via mitochondrial respiration [96]. A similar conclusion was drawn in another study: aortic tissues from CKD mice preferentially utilize oxidative phosphorylation (OXPHOS), and VSMCs stimulated with high phosphate or human uremic serum show enhanced mitochondrial respiratory capacity without significant differences in glycolysis [97]. When human coronary artery VSMCs are cultured in osteogenic medium (OM) or high-calcium phosphate medium (CaP), those treated with CaP display reduced mitochondrial respiration and glycolysis compared to OM-treated VSMCs (with enhanced mitochondrial respiration but unaltered glycolysis) [98]. 

The calcium and phosphorus sensitivity of the cell lines used in different experiments varies, and the concentrations of inorganic phosphorus and pH values set in the experiments also differ. Additionally, as shown in Table 1, whether calcium and phosphorus are used in combination is one of the key factors contributing to variations in VSMC glycolysis levels. In conclusion, current research in this field still has limitations, and more targeted experiments are urgently needed to further explore the relevant mechanisms.

#### 5.1.2. Mitochondrial Dysfunction and VSMC Glucose Metabolism in Medial Calcification

Mitochondrial dysfunction is involved in medial calcification [101,102], and multiple studies have demonstrated that glycolytic metabolic enzymes and metabolites also play roles in this process. Increased PDK4 expression in phosphate-treated VSMCs disrupts the integrity of mitochondria-associated endoplasmic reticulum membranes and impairs mitochondrial respiratory capacity [92,94]. Pyruvate, the end product of glycolysis, can be converted to lactate by LDH. Lactate induces Drp1 migration to mitochondria to enhance mitochondrial fission on one hand, and inhibits mitophagy mediated by the BNIP3 pathway or PINK1/Parkin pathway on the other hand, via activating the NR4A1/DNA-PKcs/p53 pathway and upregulating PARP1. This leads to mitochondrial structural damage, impaired respiratory function, subsequent cell apoptosis, and accelerated vascular calcification [103,104]. Additionally, upregulated PARP1 can inhibit POLG-mediated mitochondrial DNA synthesis and further exacerbate mitochondrial homeostasis imbalance by upregulating UCP2 through the PARP1/POLG signal [104].

#### 5.1.3. Advanced Glycation End Products and VSMC Glucose Metabolism in Medial Calcification

Maintenance hemodialysis patients have high basal levels of circulating advanced glycation end products (AGEs) [105], and accumulated AGEs have been reported to be associated with medial calcification of the radial artery in CKD patients [106]. In rat vascular smooth muscle cells, Nε-carboxymethyllysine (CML), a major immunogen of AGEs, increases PDK4 expression via elevated ROS, upregulates glycolysis, and accelerates calcium deposition [107]. However, another study found that AGEs significantly inhibited lactate production and glucose utilization during VSMC calcification induction. After AGE treatment, the expression levels of HK, LDH, IDH, and G6PD related to glucose metabolism were decreased, along with reduced mitochondrial respiratory capacity [108]. The impact of AGEs on VSMC glucose metabolism and the specific mechanisms deserve further investigation. In VSMCs, AGEs-induced HIF-1α activation can upregulate PDK4 and GLUT-1 expression, promoting VSMC calcification [108]. Meanwhile, this pathway can also increase LC3-II expression and decrease p62 levels, thereby enhancing VSMC autophagy, which exerts a protective effect against AGEs-induced osteogenic differentiation and calcification of VSMCs [109]. Thus, the mechanism by which AGEs promote vascular calcification is elaborate and complex.

In summary, the pathogenesis of medial calcification in the context of CKD and hemodialysis is complex and multifaceted. Studies have shown that different pathological factors can lead to divergent shifts in glucose metabolism of VSMCs. In this regard, in-depth exploration of the crosstalk between metabolic changes induced by different factors and their molecular mechanisms is expected to become an important direction for future research.

### 5.2. Lipid Metabolism

Proprotein convertase subtilisin/kexin type 9 (PCSK9) increases circulating low-density lipoprotein cholesterol (LDL-c) levels by enhancing the sorting and lysosomal delivery of cell-surface LDLRs [110]. Plasma PCSK9 is inversely correlated with glomerular filtration rate, and PSCK9 mediates media calcification induced by high inorganic phosphate levels in CKD by promoting the transition of smooth muscle cells to a calcified phenotype and releasing vesicles containing Ca^2+^ and ALP [111]. Plasma levels of the adipokine chemerin are elevated in CKD patients, and chemerin significantly reduces phosphate-induced VSMC calcification while increasing MGP expression [112]. 

### 5.3. Amino Acid Metabolism

In mice with hyperphosphatemia-induced vascular calcification, plasma concentrations of high arginine are low, and high arginine impairs NO synthesis mediated by NO synthase (NOS) isoenzymes, thereby enhancing vascular calcification [113]. H_2_S may upregulate elastin levels by inhibiting the signal transducer and activator of transcription 3/cathepsin S signaling pathway, thereby alleviating high glucose-induced vascular calcification [114].

## 6. Metabolic Crosstalk Between VSMCs and ECs During AVF Dysfunction

The relationship between metabolic reprogramming of vascular endothelial cells and AVF dysfunction is one of the current research hotspots. Endothelial cells are adjacent to VSMCs, and the metabolic crosstalk between them is also worthy of discussion. After AVF creation, increased NO secretion by endothelial cells mediated by endothelial nitric oxide synthase (eNOS) can promote VSMC relaxation to adapt to high-flow arterial blood flow; eNOS can also promote adaptive remodeling of the venous wall through anti-inflammatory, antithrombotic, and antiproliferative properties [115]. Following endothelial loss, exposure to subendothelial collagen promotes the secretion of platelet-derived microvesicles (aPMVs); aPMVs significantly reduce glycolysis, increase oxidative phosphorylation in VSMCs via the Pka-PRKAA-FoxO1 signaling pathway, and promote neointimal hyperplasia [116]. Using a co-culture model of vascular endothelial cells (VECs) and VSMCs, it was found that lactate generated by VECs acts as a signaling molecule, activating PARP1 in VSMCs and disrupting mitochondrial homeostasis via the POLG/UCP2 pathway, thereby accelerating VSMC calcification [104]. NO released by endothelial cells also plays an important role in preventing medial calcification. Both warfarin and L-NAME can promote medial calcification by impairing the function of NO released by VECs [117].

## 7. Potential Therapeutic Approaches Targeting the Metabolic Reprogramming of VSMCs in AVF Failure

In summary, metabolic alterations in VSMCs play a key role in the pathological process of AVF dysfunction. Based on this, the following section will briefly outline potential metabolic targets identified in recent studies that could be used to improve AVF dysfunction.

### 7.1. Neointimal Hyperplasia

In injured arteries, myotubularin-related protein 7 (MTMR7) expression is significantly downregulated. MTMR7 suppresses injury-induced intimal hyperplasia by inhibiting p62/mTORC1-mediated glycolysis [118]. Bergenin treatment significantly upregulates Ndufs2, an essential subunit of mitochondrial complex I, partially reverses the Warburg-like glucose metabolism pattern induced by PDGF in VSMCs, and effectively alleviates intimal hyperplasia [119]. N-Butylidenephthalide (BP) prevents AVF stenosis by activating AMPK and inhibiting mTOR phosphorylation, thereby suppressing PDGF-induced VSMC phenotypic switching and migration [120]. Propolis significantly improves lipid profiles in hypercholesterolemic rabbits, inhibits the TLR4-mediated NF-κB signaling pathway to enhance anti-inflammatory activity, and prevents neointimal hyperplasia [121]. A recent randomized controlled trial conducted among the elderly population has shown that supplementing with propolis did not result in any significant side effects [122]. However, it is important to note that some individuals with atopic constitution may experience allergic reactions after taking propolis [123]. The glutamine antagonist DONis a safe and effective drug in cancer metabolic therapy [124]. DON inhibits both upregulated glycolysis and glutaminolysis in proliferating VSMCs. JHU-083, a DON-derived prodrug, significantly reduces carotid artery ligation-induced neointima formation in mice [91].

### 7.2. Medial Calcification

U50,488H, a selective κ-opioid receptor agonist, inhibits β-GP-induced calcification of rat smooth muscle cells by reducing PFKFB3 expression and lactate content [93]. Pioglitazone, a peroxisome proliferator-activated receptor γ (PPARγ) agonist, can alleviate periostin-induced upregulation of glycolysis and downregulation of oxidative phosphorylation [125]. Serum zinc levels are significantly decreased in patients with chronic kidney disease. Zinc not only inhibits phosphate-induced vascular smooth muscle cell mineralization in a dose-dependent manner, but also attenuates the pro-calcification effects of HIF prolyl hydroxylase inhibitor (PHI), a therapeutic drug for renal anemia, including the inhibition of PDK4 expression and chondrogenic phenotype switching [126]. BGP-15 inhibits high Pi-induced upregulation of PDK4 expression and calcification of human VSMCs under both normal glucose (NG) and high glucose (HG) conditions. Importantly, BGP-15 also prevents calcium deposition in the extracellular matrix [127]. Metformin restores β-GP-impaired mitochondrial biogenesis in VSMCs via AMPK, enhances mitochondrial homeostasis, and thereby inhibits PDK4/oxidative stress-mediated apoptosis, ultimately attenuating the osteogenic phenotypic transition of VSMCs [128]. GAd, a key component in lipid metabolism, attenuates osteogenic differentiation of VSMCs by blocking PI3K/AKT and Wnt/β-catenin signaling [129].

Statins are clinically widely used lipid-regulating agents that reduce endogenous cholesterol synthesis by inhibiting HMG-CoA reductase activity. Additionally, they suppress cell apoptosis via restoring the Gas6-Axl pathway, thereby effectively preventing VSMC calcification [130]. Similarly, vitamin K2 can restore Gas6 expression, activate the downstream signaling pathways of Axl, p-Akt, and Bcl2, and block cell apoptosis, thereby alleviate VSMC calcification induced by CaCl_2_ and β-GP [131]. Additionally, vitamin K activates matrix Gla protein (MGP) via carboxylating glutamic acid residues in specific proteins, thereby inhibiting vascular calcification [132].

## 8. Conclusions and Future Directions

Metabolic reprogramming is a current research hotspot, and this review has elaborated on the role of VSMC metabolic reprogramming in key stages of AVF dysfunction. However, the AVF environment involves many stimuli, and the specific metabolic regulation mechanisms and interactions of these factors are not well understood. Meanwhile, there are still few studies on VSMC metabolism in AVF models, particularly regarding the mechanisms of contractile smooth muscle proliferation during AVF maturation and the precise metabolic changes of VSMCs in medial calcification, which all require in-depth exploration. In the future, metabolomics techniques can be applied in AVF models to further explore the regulatory role of VSMC metabolic reprogramming in AVF dysfunction, thereby identifying effective targets for translational therapy.

## Figures and Tables

**Table 1:** Summary of the effects of high-phosphate environment on VSMC metabolism in different studies.

High-Phosphate Environment Subtype	Cell Type	Metabolic Change	References
Pi (composed of a mixture of Na_2_HPO_4_ and NaH_2_PO_4_, pH 7.4), 3.5 mM	Human VSMCs	Increased PDK4 expression, elevated PDHE1α phosphorylation, reduced PDC activity, increased glucose consumption and lactate production, and a shift in glucose metabolism toward glycolysis.	[92]
10 mM β-glycerophosphate	Rat aortic VSMCs	PHD2 expression was significantly decreased, while PFKFB3 expression was significantly increased, with lactate levels elevated by 42.33% and LDH levels increased by 83.72%.	[93]
10 mM β-glycerophosphate	Rat thoracic aortic VSMCs	Mitochondrial integrity in VSMCs is impaired. The protein expression of PDK4 increases significantly at 12 h, then decreases at 48 and 72 h. The protein expression of most glycolytic genes, including GLUT1, PKM2, LDHA, and MCT4, increases in a time-dependent manner after β-GP treatment, and the metabolic reprogramming of VSMCs shifts toward the Warburg effect.	[94]
Pi (prepared as a combination of NaH_2_PO_4_ and Na_2_HPO_4_ with a ratio of 1:3, pH 7.4), 3 mM	Mice VSMCs	In VSMCs, PFKFB3 expression is increased, and intracellular lactate levels are significantly elevated.	[95]
10 mM β-glycerophosphate and 1.5 mM calcium chloride	Human aortic smooth muscle cells (HAoSMCs)	In VSMCs, basal respiration, mitochondrial ATP production, and proton leakage are increased, while spare respiratory capacity and coupling efficiency are decreased, with no changes in non-mitochondrial respiration or maximal respiration. Additionally, the capacity to oxidize glutamine and long-chain fatty acids is enhanced. However, glycolytic function, basal and glycolytic proton efflux rates in VSMCs remain unchanged, whereas non-glycolytic acidification is increased.	[96]
3.0 mM Pi	Mouse vascular smooth muscle cell (MOVAS) cell line	VSMCs exhibit a significantly higher glucose consumption rate, with increased expression levels of genes promoting pyruvate influx into mitochondria for OXPHOS, while glycolysis-related genes remain at similar levels during differentiation.	[97]
0.9 mM Na_2_HPO_4_/NaH_2_PO_4_ and 1.8 mM calcium chloride	human coronary artery SMCs	Both mitochondrial respiration and glycolysis are decreased in VSMCs.	[98]

Vascular smooth muscle cell (VSMC); Pyruvate dehydrogenase kinase 4 (PDK4); Pyruvate dehydrogenase E1 alpha subunit (PDHE1α); Pyruvate dehydrogenase complex (PDC); Prolyl hydroxylase domain-containing protein 2 (PHD2); 6-phosphofructo-2-kinase/fructose-2,6-bisphosphatase 3 (PFKFB3); Lactate dehydrogenase (LDH); Glucose transporter 1 (GLUT1); Pyruvate kinase M2 (PKM2); Monocarboxylate transporter 4 (MCT4); Adenosine triphosphate (ATP); Oxidative phosphorylation (OXPHOS).

## Data Availability

Not applicable.
